# Insecticidal Serralysin of *Serratia marcescens* Is Detoxified in M3 Midgut Region of *Riptortus pedestris*

**DOI:** 10.3389/fmicb.2022.913113

**Published:** 2022-05-31

**Authors:** Junbeom Lee, Dae-Weon Lee

**Affiliations:** ^1^Metabolomics Research Center for Functional Materials, Kyungsung University, Busan, South Korea; ^2^Department of SmartBio, Kyungsung University, Busan, South Korea

**Keywords:** insect symbiosis, serralysin, detoxification, *Riptortus pedestris*, *Serratia marcescens* Db11

## Abstract

*Riptortus pedestris* insect indiscriminately acquires not only the symbiotic bacterium *Burkholderia insecticola*, but also entomopathogens that are abundant in the soil *via* feeding. However, it is unclear how the host insect survives oral infections of entomopathogens. A previous study suggested that serralysin, a potent virulence factor produced by *Serratia marcescens*, suppresses cellular immunity by degrading adhesion molecules, thereby contributing to bacterial pathogenesis. Here, we observed that *S. marcescens* orally administered to *R. pedestris* stably colonized the insect midgut, while not exhibiting insecticidal activity. Additionally, oral infection with *S. marcescens* did not affect the host growth or fitness. When co-incubated with the midgut lysates of *R. pedestris*, serralysin was remarkably degraded. The detoxification activity against serralysin was enhanced in the midgut extract of gut symbiont-colonizing insects. The mRNA expression levels of *serralysin* genes were negligible in M3-colonizing *S. marcescens*. M3-colonizing *S. marcescens* did not produce serralysin toxin. Immunoblot analyses revealed that serralysin was not detected in the M3 midgut region. The findings of our study suggest that orally infected *S. marcescens* lose entomopathogenicity through host-derived degrading factors and suppression of serralysin.

## Introduction

*Serratia* species is frequently found in the midgut bacterial flora of almost all hematopoietic insects, such as mosquitoes and *Rhodnius* species (Demaio et al., 1996; Straif et al., 1998; Gonzalez-Ceron et al., 2003; Azambuja et al., 2004). The gram-negative bacterium, *Serratia marcescens*, acts as an opportunistic pathogen in a wide range of animals, including humans and insects (Raymann et al., 2018). *Serratia marcescens* is an entomopathogen that causes bacteremia in the insect hemolymph, resulting in rapid death (Grimont and Grimont, 1978; Chadwick et al., 1990). The gut commensal *S. marcescens* promotes mosquito permissiveness to arboviruses and facilitates arboviral infection *via* a secreted protein *Sm*Enhancin (Wu et al., 2019). In the gut of a cockroach, *Gromphadorhina portentosa* (Madagascar), *S. marcescens* not only produces active molecule(s) with potent antibacterial properties but also exhibits anti-amoebic effects (Akbar et al., 2018). This entomopathogen produces a virulence factor, called serralysin, which shows insecticidal activity *via* hemolymph bleeding and inhibition of immune cell adhesion (Tambong et al., 2014; Ishii et al., 2014a,b). Notably, serralysin degrades the humoral immune factors in insects (Lee et al., 2017a). Serralysin is a conserved metalloprotease among various bacterial species and requires both zinc and a divalent cation for maximal catalysis (Bode et al., 1993; Belas et al., 2004). The entomopathogen *Xenorhabdus* has a serralysin-type metalloenzyme called PrtA (Massaoud et al., 2011). *Proteus* and *Photorhabdus* species also have the serralysin homologs ZapA and AprA, respectively, which inhibit antibacterial activity by destroying antimicrobial peptides, such as defensin and cecropin (Belas et al., 2004; Cabral et al., 2004). The serralysin family is grouped in the metzincin metalloprotease superfamily which is characterized by the zinc-binding motif (HEXXHXXGXXHZ; Bode et al., 1993; Salamone and Wodzinski, 1997).

*Riptortus pedestris*-*Burkholderia insecticola* symbiosis is a powerful model system for analyzing molecular cross-talks. The bean bug, *R. pedestris* (Hemiptera: Alydidae), possesses a specialized symbiotic organ in the posterior region of the midgut (referred to as the M4 crypt), where numerous crypts harbor specific gut symbionts of the β-proteobacterial genus, *Burkholderia* (Kikuchi et al., 2005). In this symbiosis model, *Burkholderia*-colonized insects (Sym-insects) and insects without gut symbionts (Apo-insects) can be generated in the laboratory depending on oral infection of *Burkholderia* cells, respectively (Lee et al., 2017b, 2019, 2022). However, the gut immune responses of *R. pedestris* to exogenous microbes have not yet been clearly elucidated. Using this model, we recently observed that orally infected entomopathogenic *S. marcescens* cells are resistant to the antimicrobial peptide, trialysin, produced in the salivary glands of *R. pedestris* (Lee et al., 2017a). However, it is unclear how the pathogenicity of *S. marcescens* is eventually suppressed in the midgut.

In this study, orally infected *S. marcescens* were found to colonize the midgut of *R. pedestris*, but did not exhibit any insecticidal activity against the host. Serralysin was markedly degraded by host-derived factors present in the M1 midgut region. The M3-colonizing *S. marcescens* cells did not produce serralysin. These results suggest that orally infected *S. marcescens* lose insecticidal activity through expression of host-derived degrading factors and suppression of serralysin molecules.

## Materials and Methods

### Insect Rearing and *Serratia marcescens* Inoculation

*Riptortus pedestris* was maintained in an insectary at 28°C under a long-day cycle of 16 h light and 8 h dark, as described previously (Lee et al., 2020). The nymphs were reared in clean plastic containers with soybean seeds and distilled water containing 0.05% ascorbic acid. The non-pigmented *S. marcescens* strain Db11 was cultured to mid-log phase at 37°C in the Luria-Bertani medium (BD Difco, United States; Lee et al., 2017a). Newly molted second-instar nymphs were inoculated with *S. marcescens* Db11 cells at a concentration of 10^7^ cells/ml.

### Fitness Measurement

To compare the effect of *S. marcescens* oral infection on the host fitness, nymphs were reared until adulthood and assessed for various fitness parameters, such as emergence rate, survival rate, dry weight, and body length (Lee et al., 2017b). Emergence was monitored daily by inspecting the late-fifth-instar nymphs and counting the number of newly molted adult insects. Survival rates were examined from the second instar to the adult stage when *S. marcescens* was orally infected. For dry weight measurements, adult males were immersed in acetone for 5 min and then completely dried in an oven at 70°C for 10 min.

### Purification of Serralysin

Serralysin was purified from bacterial cultures according to a previous report (Lee et al., 2017a). Briefly, bacteria were grown at 30°C to an optical density at 600 nm (OD_600_) of 3.0 in the brain heart infusion medium (BD Difco, United States). The supernatant was filtered through a 0.2 μm pore filter (Whatman, United States), ultrafiltered, and replaced with buffer A (50 mM Tris-HCl, pH 8.0) using a Vivaspin 3,000 Da filter (10,000 × *g*, 30 min, 4°C; Sartorius, United States). The solutions were loaded onto a Mono-Q column equilibrated with buffer A (Metabolomics Research Center for Functional Materials, Kyungsung University; Agilent Technologies 1260 Infinity LC System, United States). The column was eluted with 30 ml of a linear gradient of NaCl (0–1 M) in buffer A at a flow rate of 0.5 ml/min. Active fractions were collected, N-terminally sequenced, and used for further experiments (Lee et al., 2017a).

### Administration of Serralysin and Measurement of Survival Rate

Purified serralysin was sequentially diluted to a concentration of 1 μg/μl. Each serralysin solution at a different dose (2 μl aliquot) was orally infected or systemically injected into the joint of the hind leg that connects to the 3-day-old adult male, *R. pedestris*. The survival rate was monitored from 1 d after the systemic injection.

### Preparation of Midgut Lysates From Apo- and Sym-Insects

Midguts from 10 male *R. pedestris* adults were collected in 500 μl of 10 mM phosphate buffer (PB; pH 7.0) containing a protease inhibitor cocktail (Sigma Aldrich, United States). The collected midguts were homogenized using disposable plastic pestles (SciLab Korea, South Korea). After centrifugation at 10,000 × *g* for 15 min at 4°C, the supernatant was ultrafiltered and replaced with buffer A (50 mM Tris–HCl, pH 8.0) using a Vivaspin 3,000 Da filter (10,000 × *g*, 30 min, 4°C; Sartorius, Germany). After buffer replacement, the solution was collected and used immediately for further experiments. The supernatant was heat-treated at 90°C for 5 min to inactivate the serralysin-degrading proteins and centrifuged at 20,000 × *g* for 10 min at 4°C. Bovine serum albumin was used for protein quantification of midgut lysates, and the native protein concentration was determined using the Bradford assay (Kruger, 2009).

### Immunoblot Analyses

For immunoblot analyses, proteins from M3 lysate or hemolymph were separated on a 10% sodium dodecyl sulfate-polyacrylamide gel and transferred to a polyvinylidene difluoride membrane (Millipore, United States) using an electro-transfer blotter (Lee et al., 2017a). The membrane was blocked with 10% skim milk in Tris-buffered saline with Tween 20 (TBST) buffer (50 mM Tris-HCl, 150 mM NaCl, and 0.02% Tween 20) for 1 h and washed six times with the TBST buffer. After blocking, the membranes were incubated at room temperature for 1 h with the anti-rabbit serralysin antibody (dilution factor 1:5,000) in TBST containing 5% skimmed milk (Lee et al., 2017a). After washing with TBST six times, the membrane was incubated with horseradish peroxidase (HRP)-conjugated goat anti-rabbit IgG secondary antibody (dilution factor 1:10,000; Santa Cruz, United States) for 30 min. The membranes were washed seven times with TBST and visualized using HRP color development solution in accordance with the manufacturer’s instructions.

### Isolation of Midgut-Colonizing *Serratia marcescens* Cells

M3 midguts were dissected from 10 fifth-instar nymphs and collected in 100 μl of 10 mM PB. M3 midguts were cut several times with fine scissors to break the midgut crypts. One milliliter of 10 mM PB was added to the midgut fragments by gentle pipetting. The solution was then filtered through a 5.0 μm pore to remove the midgut tissues. Isolated *S. marcescens* cells were gently washed with 10 mM PB and collected by centrifugation at 2,000 × *g*. The bacterial cell number was estimated using a hemocytometer.

### Quantitative Real-Time Polymerase Chain Reaction

Total RNA was isolated from midgut-colonizing or cultured *S. marcescens* cells (OD_600 nm_ = 1.0) using the TRIzol reagent (Invitrogen, United States) and RNeasy mini kit (Qiagen, United States), according to the manufacturer’s recommendations. Thereafter, 500 ng of total RNA was converted into cDNA using TOPscript RT DryMix containing oligo-dT primers (Enzynomics, South Korea). The synthesized cDNAs were diluted 20-fold, and qRT-PCR was performed on a QuantStudio™3 Real-Time PCR System (Thermo Fisher Scientific Inc., United States). The PCR conditions were as follows: 95°C for 10 min, followed by 40 cycles of 95°C for 10 s, 60°C for 15 s, and 72°C for 20 s. The primer sets used for qRT-PCR of *serralysin* genes are listed in Table 1. The comparative *C_T_* (∆∆*C_T_*) method was used to calculate the relative gene expression levels based on the 16S rRNA of *S. marcescens* cells as an internal control gene (Wilf et al., 2011). All analyses were performed using the QuantStudio™ Design & Analysis Software ver 1.5.2 (Thermo Fisher Scientific Inc.,).

**Table 1:** Primer sets used for qRT-PCR in this study.

Primer name	Primer sequence (5′→3′)
Serralysin 1_F	ATCACCTTCACCGAAGTGG
Serralysin 1_R	TGCTTGACGTTGGACTGG
Serralysin 2_F	ATGAGAACGAGTACGGTCG
Serralysin 2_R	CTTTAAAGTCCTGCCCGGT
16S rRNA _F	GGCCTTCGGGTTGTAAAGTC
16S rRNA _R	GCTTTACGCCCAGTCATTC

### Statistical Analysis

Statistical significance of the data was determined using an unpaired Student’s *t*-test, as provided in GraphPad Prism (ver. 8.0; GraphPad Software, United States).

## Results

### Midgut-Colonizing *Serratia marcescens* Cells Did Not Affect Host Fitness

**Figure 1:**
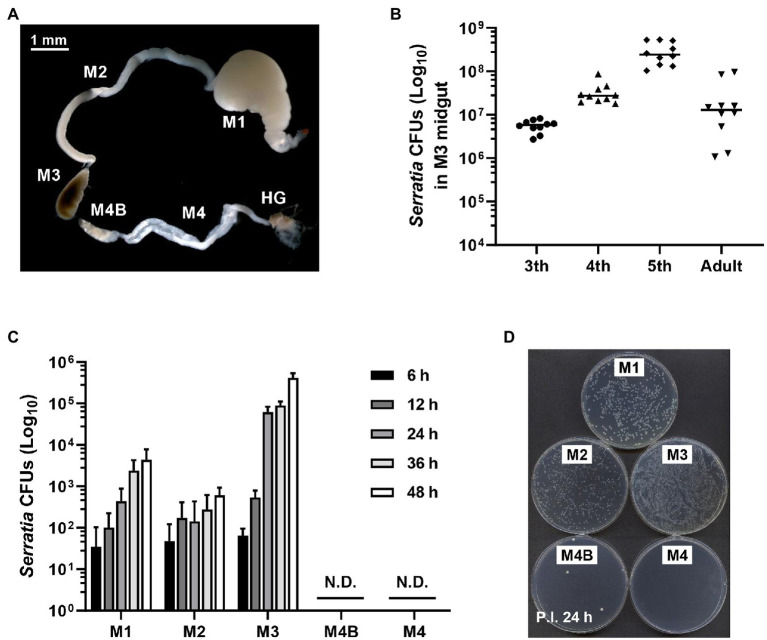
Colonization of orally infected *Serratia marcescens*. **(A)** Midgut (M1, M2, M3, M4B, and M4) and hindgut (HG) regions of the *Riptortus pedestris* insect. **(B)** Infection density of *S. marcescens* at third, fourth, fifth-instar stages and male adults. Means and standard deviations (SDs; *n* = 10) are shown as columns and error bars, respectively. **(C)** Colony-forming unit (CFU) quantification of infection densities of *S. marcescens* Db11 cells at 6, 12, 24, 36, and 48 h after inoculation. Horizontal lines in the graph indicate the mean values of *S. marcescens* CFUs. Error bars indicate the SD of the mean (*n* = 10). (N.D., not detected). **(D)** Colonies on agar plate of *S. marcescens* cells at an early stage of infection.

Previous studies have shown that *S. marcescens* Db11 strain is highly pathogenic to *Drosophila melanogaster* and *Apis mellifera* when they are systemically injected with this bacterium (Flyg et al., 1980; Kurz et al., 2003; Raymann et al., 2018). However, orally infected *S. marcescens* Db11 cells can colonize the *Drosophila* midgut without any virulence (Nehme et al., 2007). Based on these reports, we hypothesized that orally infected *S. marcescens* cells could colonize the midgut of *R. pedestris*. The bean bug adults have five different midgut regions (Ohbayashi et al., 2015), M1, M2, M3, M4B, and M4. We examined the colony-forming units (CFUs) of *S. marcescens* in each midgut region at the indicated times (Figure 1A). *Serratia marcescens* cells were only detected in M1–M3 regions 2 days post-inoculation (Figures 1C,D). These results suggest that orally infected *S. marcescens* cells can pass through the digestive tract, such as the foregut region and colonize the midgut. The beneficial symbiont, *B. insecticola*, colonized M4B and M4 midgut regions (Figures 1C,D). This result is consistent with the previous report that no microorganism or color pigment can pass through M4B region except *Burkholderia* (Ohbayashi et al., 2015). When we examined the population of orally infected *S. marcescens* cells in M3 midgut region, the titers of *S. marcescens* were increased steadily during nymphal development and remained at constant levels of around 10^7^ CFUs per individual in adulthood (Figure 1B).

**Figure 2:**
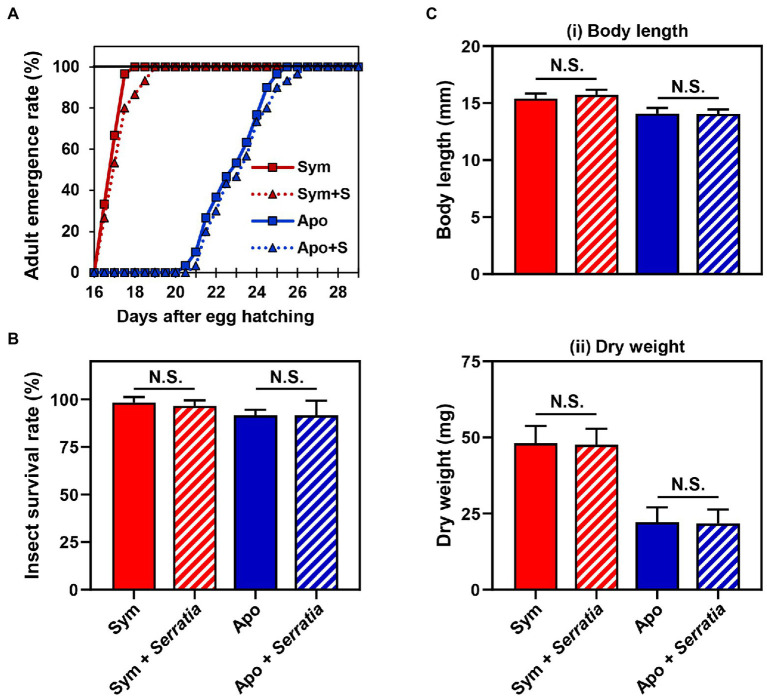
Effects of orally infected *Serratia marcescens* on *Riptortus pedestris.* Comparison of the **(A)** developmental rate, **(B)** survival rate, and **(C)** fitness parameters of male *R. pedestris* insects after oral infection with *S. marcescens*. The fitness effects of each group were analyzed by measuring the (i) body length and (ii) dry weight of male *R. pedestris* 5 days after adult emergence. Error bars indicate the SD of the mean (*n* = 30). (N.S., not significant).

Based on the fact that *Burkholderia* gut symbionts positively affect the host fitness and immune responses (Kim et al., 2015a; Lee et al., 2017b), we investigated if orally infected *S. marcescens* is a potent entomopathogen that affects the survival rate and growth of the host. Furthermore, we speculated that Sym-insects with *Burkholderia* gut symbionts would be more resistant to orally infected *S. marcescens* than Apo-insects without symbionts. To investigate the effects of *S. marcescens* cells on either Sym- or Apo-insects, the adult emergence rates of *R. pedestris* were estimated after oral infection with cultured *S. marcescens* cells. Orally infected *S. marcescens* did not affect the growth of *R. pedestris* (Figure 2A). When the survival rates between Sym- and Apo-insects were compared, all second nymphs infected with *S. marcescens* were survived until adulthood (Figure 2B). Evaluation of body length and dry weight showed that orally infected *S. marcescens* did not have any effect on the host, regardless of the presence of symbiotic *Burkholderia* cells (Figure 2C). These results suggest that orally infected *S. marcescens* can colonize the midgut region without affecting host fitness.

### Serralysin Is Specifically Degraded by the Midgut Lysate

**Figure 3:**
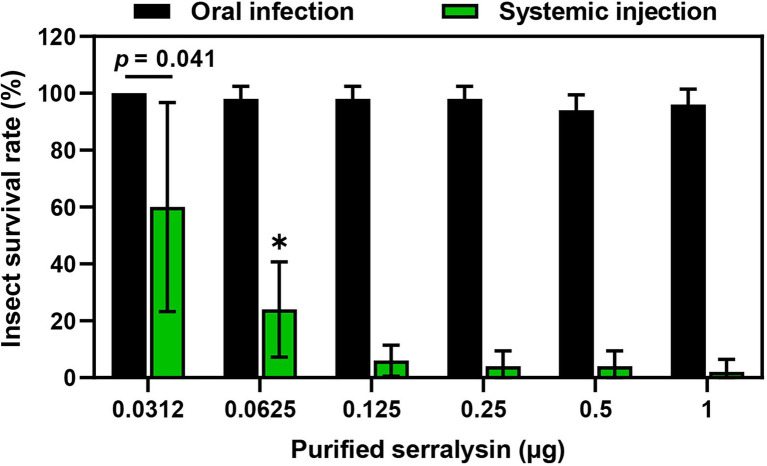
Insecticidal activity of serralysin. Purified serralysin was orally administered and systemically injected into the joint of the hind leg, which connects to the thorax of the insect. Survival was monitored 1 day after systemic injection. Data are expressed as the mean ± SD (*n* = 10) and are representative of five independent experiments. Asterisks indicate the significant differences between groups (^*^p < 0.01; unpaired *t*-test).

Serralysin metalloprotease is the major toxic substance of *S. marcescens* in host insects (Ishii et al., 2014a). However, our results showed that oral infection with *S. marcescens* did not have any adverse effects on development of *R. pedestris* (Figure 2). These results indicate that orally infected *S. marcescens* does not show any insecticidal activity during the colonization of *S. marcescens* cells in insect gut. Therefore, to compare the insecticidal activity of serralysin based on the infection routes, the survival rate was investigated by oral infection or systemic injection of serralysin purified from the bacterial culture medium. Only systemic injection of serralysin showed insecticidal activity in a dose-dependent manner whereas all tested insects were survived after oral infection (Figure 3). The amino acid sequence of purified serralysin was identified as A-A-T-T-G-Y-D-A-V-D, corresponding to the 17th to 26th amino acids of serralysin 1 (Supplementary Figure 1).

**Figure 4:**
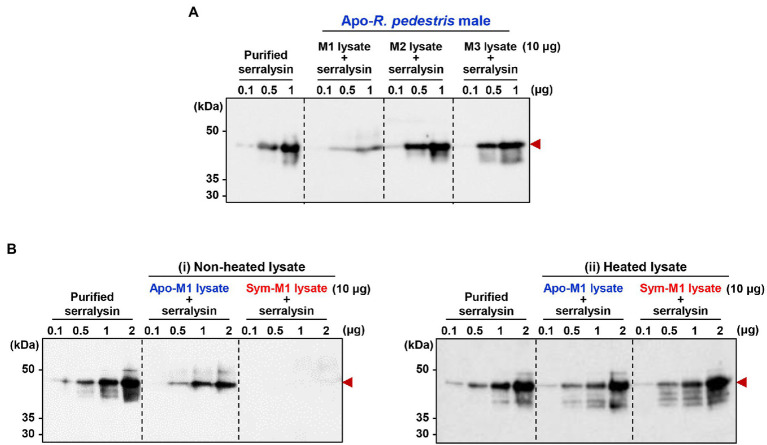
Inactivation of serralysin by midgut lysates. **(A)** Immunoblot patterns after co-incubation of purified serralysin with M1, M2, and M3 midgut lysates of insects without gut symbionts (Apo-insects). **(B)** Immunoblot patterns after co-incubation of purified serralysin with M1 midgut lysates of Apo- and *Burkholderia*-colonized insects (Sym-insects). M1 extracts were prepared (i) without and (ii) with heat treatment, respectively. The reaction conditions were as follows: In total, 10 μg of each midgut lysate was incubated with different concentrations of purified serralysin for 1 h at 28°C. Arrowheads indicate the bands of purified serralysin.

It is questionable how host insects are alive when they are orally infected with insecticidal serralysin. Our previous study provided a biochemical evidence that the antimicrobial peptide trialysin in *R. pedestris* salivary fluid was specifically hydrolyzed by serralysin, leading to the loss of its antimicrobial activity (Lee et al., 2017a). Based on these results, we hypothesized that the insecticidal activity of serralysin would be diminished during digestive tract passage and colonization by unknown factors, thereby facilitating host survival. To prove this hypothesis, purified serralysin was co-incubated with three midgut extracts of Apo-insects, and the activation of serralysin was investigated with immunoblot analyses. Purified serralysin was gradually degraded by the midgut extract in a dose-dependent manner (Figure 4). This result strongly suggests that a host-derived factor capable of degrading serralysin exists in the M1 midgut region (Figure 4A). To determine if the serralysin-degrading activity is derived from the hosts, Apo- or Sym-insects, extracts from the M1 midgut of both hosts were co-incubated with serralysin. Purified serralysin was completely removed by the M1 lysate of Sym-insects, but remained in the M1 lysate of Apo-insects (Figure 4B-i). Moreover, when the supernatant of heat-treated Sym- and Apo-M1 lysates was added to purified serralysin, the host-derived serralysin-degrading factor lost its biological activity (Figure 4B-ii). This result revealed that the serralysin-degrading factor in the M1 lysate is related to proteinous molecules.

### M3-Colonizing *Serratia marcescens* Cells Do Not Produce Serralysin

**Figure 5:**
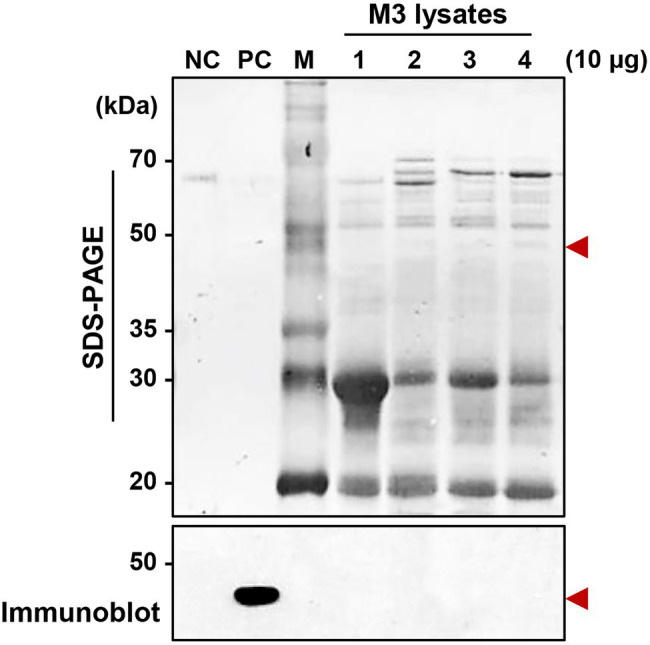
Detection of serralysin in the M3 midgut region of *Riptortus pedestris*. Sodium dodecyl sulfate-polyacrylamide gel electrophoresis (SDS-PAGE; upper layer) and immunoblot (bottom layer) of M3 lysate after oral infection of male Sym insects with *S. marcescens*. *Serratia marcescens* cells (1.0 × 10^7^ CFUs/ml) were orally infected into 3-day-old adult male *R. pedestris* insects. After infection for 48, 72, and 96 h, M3 tissues were collected from the insects, and the induction of serralysin was analyzed using specific antibodies. Arrowheads indicate the bands of purified serralysin. Negative control (NC): bovine serum albumin (BSA; 0.5 μg); positive control (PC): purified serralysin (0.5 μg); M: ladder marker. Columns 1, 2, 3, and 4 indicate the naïve M3, *S. marcescens* colonized-M3 (48 h post-inoculation (P.I.)), P.I. 72 h, and P.I. 96 h, respectively.

Although a serralysin-degrading factor from *R. pedestris* existed in M1 region (Figure 4), orally infected *S. marcescens* cells still colonized the M3 midgut region (Figure 1). To understand the host-gut microbe interactions in M3 region, we investigated if serralysin was detected in M3 tissue. Serralysin was not detected in the M3 tissue (Figure 5). This result provides the possibility that *S. marcescens* cells in M3 region do not produce serralysin during colonization of the host gut. Based on this result, we investigated that serralysin expression in M3 region can be maintained at a low level or completely suppressed. Genome analysis based on the National Center for Biotechnology Information (NCBI) public database showed that two *serralysin* genes were present in *S. marcescens* Db11. The nucleotide and putative amino acid sequences of *serralysin* 1 and *serralysin* 2 showed a similarity of 66.5 and 59.0%, respectively (Supplementary Figure 1). In addition, serralysin 1 and 2 had a catalytic domain related to metzincin at the N-terminus region (data not shown). The phylogenetic analysis revealed that serralysin 1 and serralysin 2 formed independent clades, respectively (Supplementary Figures 2, 3).

**Figure 6:**
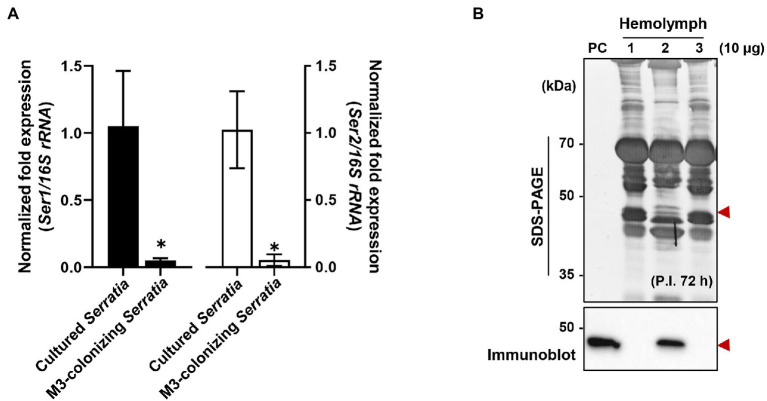
Suppression of serralysin production in M3-colonizing *Serratia marcescens* cells. **(A)** The mRNA expression levels of *serralysin* genes in cultured *S. marcescens* and M3-colonizing *S. marcescens*. The expression levels of *serralysin* genes were normalized to the expression levels of each cultured *S. marcescens*, which was set as 1. Data are expressed as the mean ± SD (*n* = 4). (^*^p < 0.01; unpaired *t*-test). The Genbank accession numbers of protein sequences are as follows: *serralysin* 1 (CDG14870.1) and *serralysin* 2 (CDG11942.1). **(B)** SDS-PAGE (upper layer) and immunoblot (bottom layer) of whole-body hemolymph after systemic injection of *S. marcescens* into male Sym insects. *Serratia marcescens* cells (1.0 × 10^5^ CFUs) systemically injected into 3-day-old adult male *Riptortus pedestris* insects for immune induction. After injection for 3 h at 28°C, immune-challenged hemolymph was collected from the insects, and induction of serralysin was analyzed using specific antibodies. Arrowheads indicate the bands of purified serralysin. PC: purified serralysin (0.5 μg). Columns 1, 2, and 3 indicate phosphate-buffered saline (PBS) injection, cultured *S. marcescens* injection, and M3-colonizing *S. marcescens* injection, respectively.

When comparing mRNA expression levels between serralysin genes in cultured *S. marcescens*, no difference in the expression level was found (Supplementary Figure 4). The transcriptional levels of *serralysin* genes were compared between *in vitro*-cultured *S. marcescens* and M3-colonizing *S. marcescens* cells. The expression levels of *serralysin* genes in M3-colonizing *S. marcescens* cells were significantly lower than those of *in vitro*-cultured *S. marcescens* (Figure 6A). Under systemic injection, M3-colonizing *S. marcescens* cells did not produce serralysin whereas *in vitro*-cultured *S. marcescens* cells secreted serralysin (Figure 6B).

## Discussion

In a previous study, we demonstrated that serralysin, a toxin produced by *S. marcescens* cells, neutralized the antimicrobial activity of trialysin in the salivary of *R. pedestris*, resulting in suppressing the host immune response (Lee et al., 2017a). When systemically inoculated into the host, serralysin exhibited insecticidal activity (Figure 3). Based on this result, we hypothesized that the serralysin toxin of orally infected *S. marcescens* could kill the host insect. However, orally infected *S. marcescens* cells were found only to colonize the midgut without insecticidal activity (Figure 1). These results led to the idea that orally infected *S. marcescens* cells may have been converted into non-pathogenic microorganisms and then lost their insecticidal activity.

Insect detoxification systems inactivate toxic substances, greatly reducing biological effects by entomopathogenic microorganisms (Hillyer, 2016). In the insect midgut, the mechanism of resistance against toxic substances is composed of complex responses of the defense system, including alteration of toxin-binding proteins (Bravo et al., 2011), sequestration of the toxin by lipophorin (Ma et al., 2012), increased production of primary enzymes (Tartar et al., 2009; Grizanova et al., 2019; Wu et al., 2020), and antioxidant activity (Dubovskiy et al., 2008). These biochemical reactions require various enzymes, such as esterases, uridine diphosphate-glycosyltransferases, cytochrome P450s, glutathione-S-transferases, and monooxygenases (Ramsey et al., 2010; Feng et al., 2021). These enzymes play important roles in the detoxification of toxic substances, such as insecticides and plant secondary metabolites (Li et al., 2007). In this study, we revealed that a host-derived factor that can degrade serralysin is present in the M1 midgut region of *R. pedestris* (Figure 4A). The serralysin-degrading activity was completely lost after heat treatment (Figure 4B). Taken together, the host-derived factors for degrading serralysin are proteinous molecules, such as detoxifying enzymes or their complexes. As serine proteases, such as trypsin and chymotrypsin, have a serine hydroxyl residue at the active site and are involved in regulation of enzyme activity, they were candidates for host-derived serralysin-degrading factors (Aiyappa and Harris, 1976). The proteolytic activity of metalloproteases including serralysin was completely inhibited by EDTA, a chelating agent for divalent metal cations (Kida et al., 2007; Ishii et al., 2014a,b). The inhibitor screening for serralysin metalloprotease can be a good strategy to identify host-derived factors.

Orally infected *S. marcescens* cells did not produce serralysin during colonization of the midgut. Transcriptome analysis by RNA sequencing of both gut-colonizing bacteria and cultured bacteria showed that the expression patterns of several genes were completely different (Ohbayashi et al., 2019). When differentially expressed genes were classified into clusters of orthologous groups of the NCBI public database, the expression levels of most genes involved in cell motility, secretion, vesicular transport, and extracellular structures were downregulated in the gut-colonized bacteria (Baumann, 2005; McCutcheon and Moran, 2012; Ohbayashi et al., 2019). Among them, Type II and type IV secretion systems are involved in the extracellular transport of cell wall-degrading enzymes and endotoxins (Sandkvist, 2001; Murdoch et al., 2011; Gallique et al., 2017; Ohbayashi et al., 2019). There are changes in gut-colonizing bacteria, such as small size, increased stress sensitivity, loss of motility, and alteration of cell surfaces (Kim et al., 2013, 2015b; Ohbayashi et al., 2019). Based on these results, we investigated that the gene expression pattern of M3-colonizing *S. marcescens* would be different from that of free-living *S. marcescens*. As expected, the transcriptional and translational expression levels of *serralysin* genes dramatically decreased in M3-colonizing *S. marcescens* (Figure 6). This explained the reason the insecticidal serralysin was not found in M3 tissue.

The previous report that lipopolysaccharide (LPS) components are associated with the insecticidal activity of *S. marcescens* may provide a molecular evidence that M3-colonizing *S. marcescens* cannot produce serralysins. In general, LPS is a requisite component for maintaining the physiological properties, such as permeability and biofilm formation (Herlax et al., 2005; Ishii et al., 2014b; Hathroubi et al., 2016). The LPS layer comprises three regions: lipid A moieties, core oligosaccharides, and O-antigen polysaccharides. Among them, the O-antigen residue is highly associated with bacterial pathogenicity. The *waaE* mutant of *Aeromonas hydrophila* which has an O-antigen-deficient LPS is avirulent and loses the ability to stimulate the pro-phenoloxidase system in *Tenebrio molitor* larvae (Noonin et al., 2010). Enterohemorrhagic *Escherichia coli* O157 requires an LPS O-antigen for effective insecticidal activity against *Bombyx mori* (Miyashita et al., 2012). Similarly, the *wecA* gene of *S. marcescens* which is involved in the biosynthesis of LPS O-antigen is an important factor for the apoptotic death of host immune cells in *B. mori* (Ishii et al., 2012). The culture supernatant of the *S. marcescens wecA* mutant exhibits reduced hemolymph bleeding activity compared to that of the wild-type strain (Ishii et al., 2014b). In *S. marcescens*, rough-type LPS lacking O-antigen components induced significantly low hemolytic activity (Poole and Braun, 1988; Kurz et al., 2003). Symbiotic *Burkholderia* colonizing the M4 crypt lacks an O-antigen residue in *R. pedestris* (Kim et al., 2015b). These molecular evidences suggest the possibility that M3-colonizing *S. marcescens* cells may lack the LPS O-antigen.

In summary, we revealed that serralysin produced by orally infected *S. marcescens* cells was degraded by the host-derived factor present in the M1 tissue of *R. pedestris*. In addition, *S. marcescens* cells colonized in the M3 region did not produce the entomopathogenic serralysin. This study will provide insight into how orally infected entomopathogenic bacteria can colonize the host midgut without insecticidal activity and the molecular interactions between them in the gut.

## Data Availability Statement

The original contributions presented in the study are included in the article/Supplementary Material, further inquiries can be directed to the corresponding authors.

## Author Contributions

JL and D-WL conceived and designed the study and wrote the manuscript. JL performed the experiments and analyzed the data. All authors contributed to the article and approved the submitted version.

## Funding

This research was supported by the Basic Science Research Program through the National Research Foundation of Korea (NRF) and funded by the Ministry of Education (NRF-2020R1I1A1A0106704312). This research was supported by a grant from the Korea Basic Science Institute (National Research Facilities and Equipment Center) and funded by the Ministry of Education (2019R1A6C1010044).

## Conflict of Interest

The authors declare that the research was conducted in the absence of any commercial or financial relationships that could be construed as a potential conflict of interest.

## Publisher’s Note

All claims expressed in this article are solely those of the authors and do not necessarily represent those of their affiliated organizations, or those of the publisher, the editors and the reviewers. Any product that may be evaluated in this article, or claim that may be made by its manufacturer, is not guaranteed or endorsed by the publisher.
