# Supplementary figures and images for: Insecticidal Serralysin of Serratia marcescens Is Detoxified in M3 Midgut Region of Riptortus pedestris
Source: Front Microbiol. 2022 May 31;13:913113. doi: 10.3389/fmicb.2022.913113 (PMC9197470; doi:10.3389/fmicb.2022.913113)

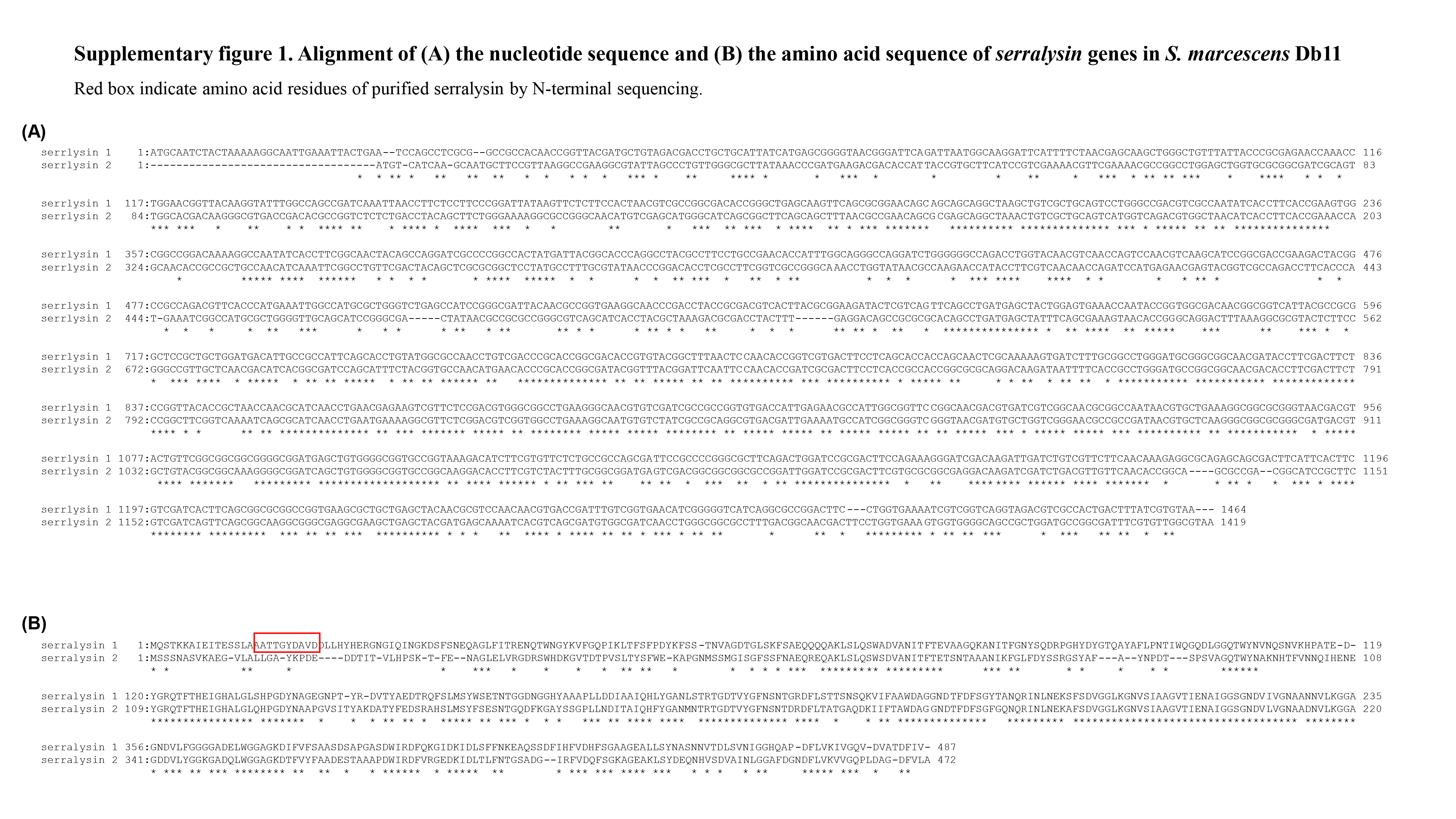

Supplement: Supplementary file 1 [file Image_1.JPEG]

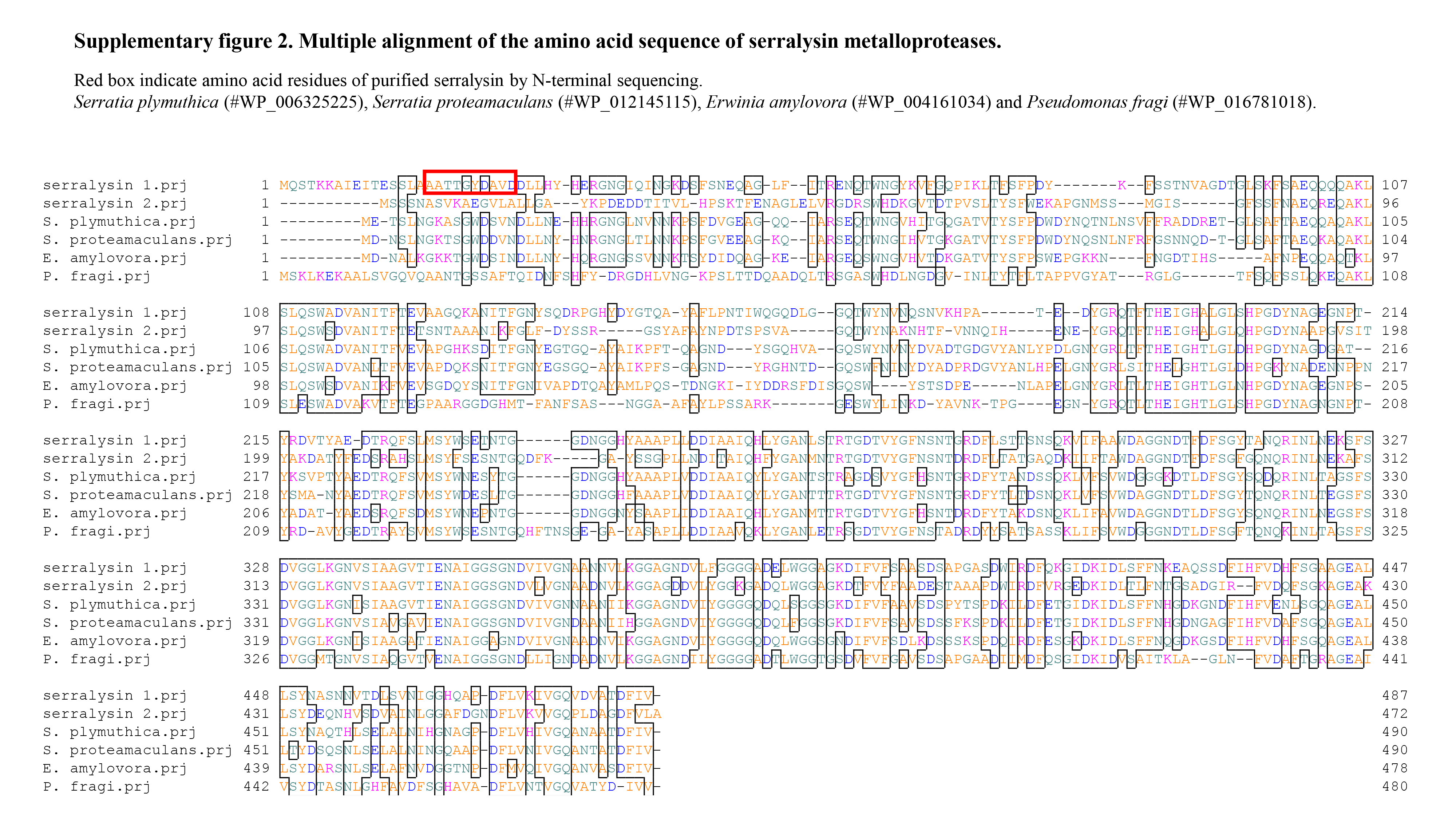

Supplement: Supplementary file 2 [file Image_2.JPEG]

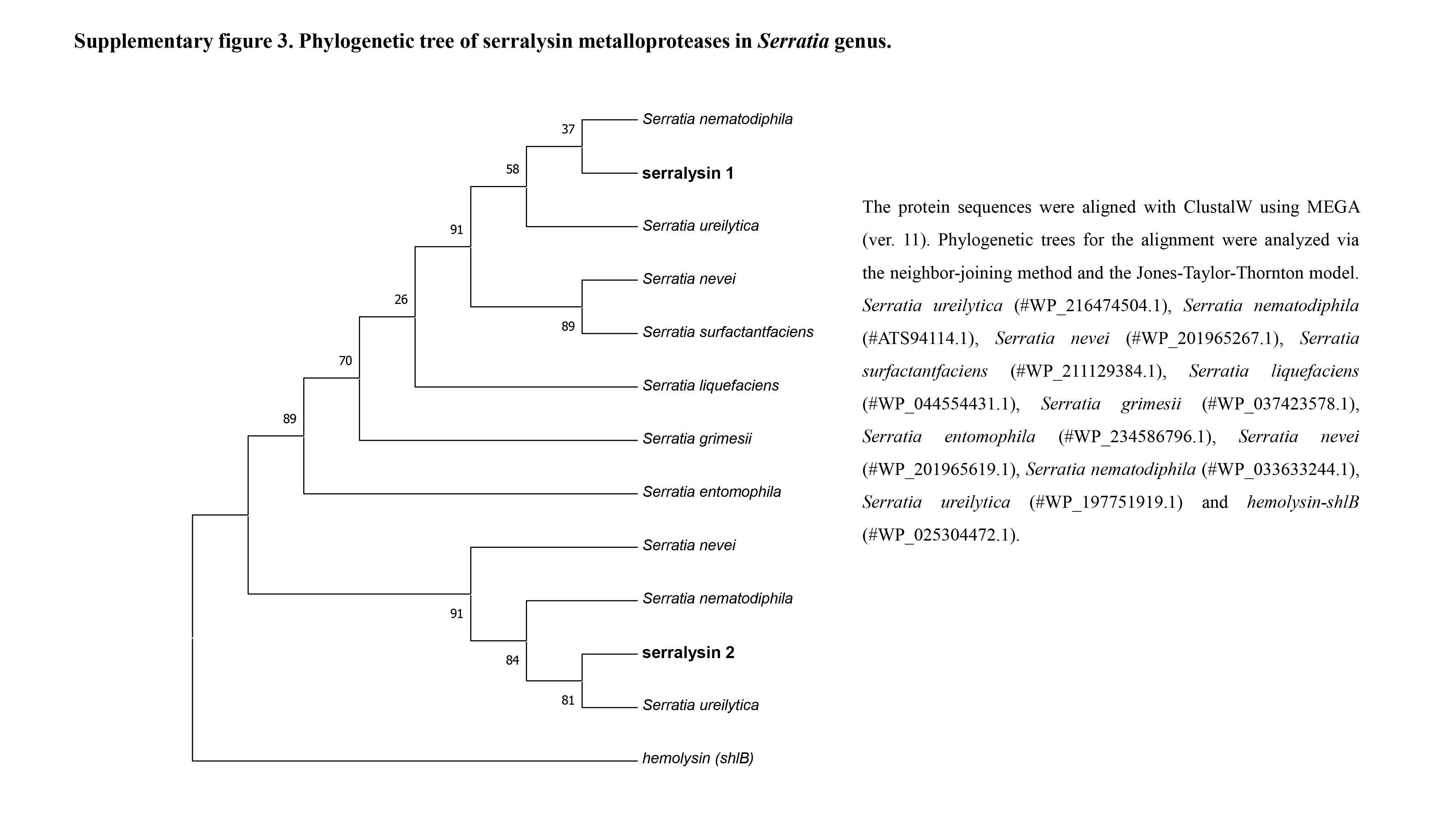

Supplement: Supplementary file 3 [file Image_3.JPEG]

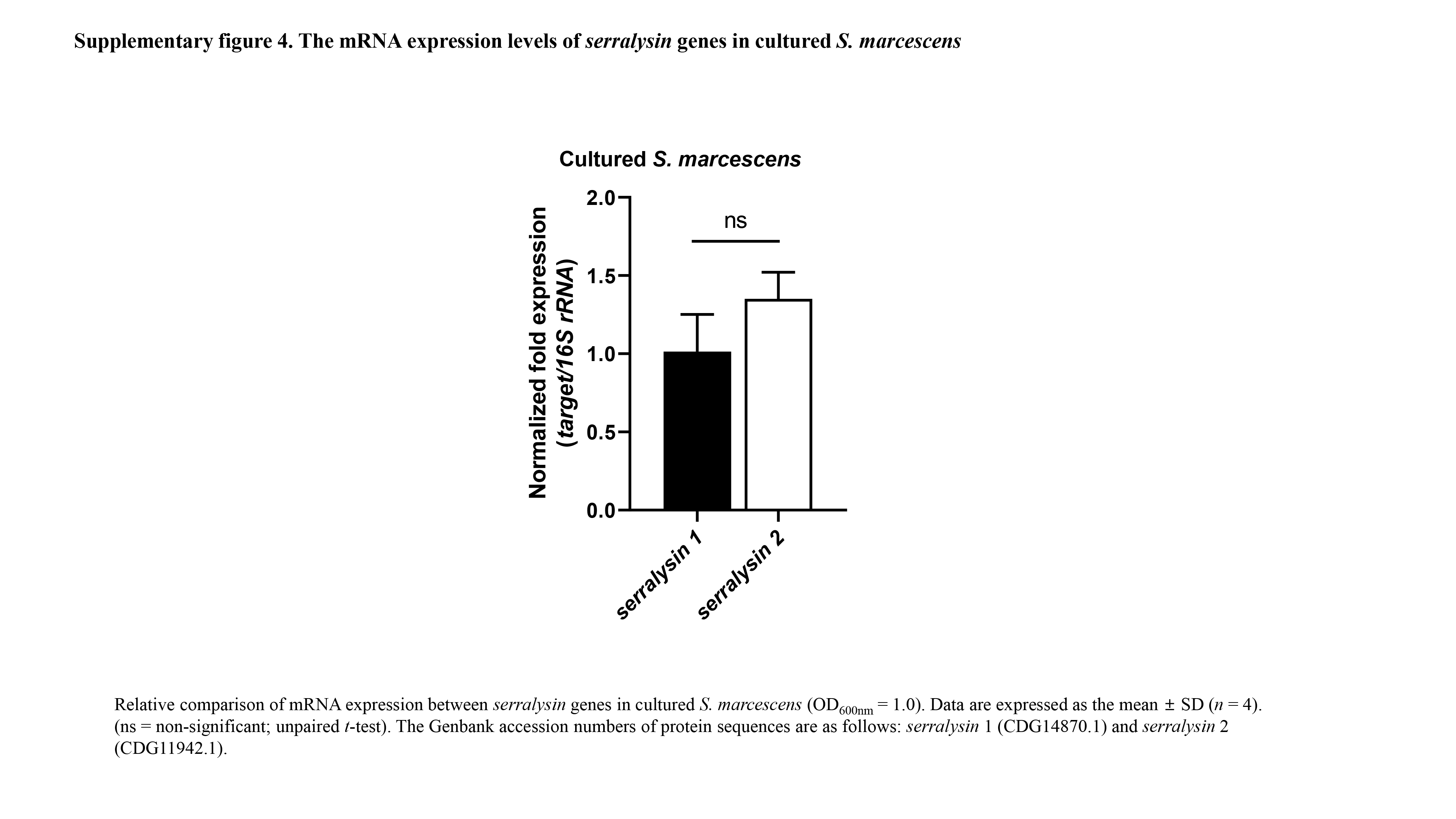

Supplement: Supplementary file 4 [file Image_4.JPEG]
